# Clustering of Pulmonary Tuberculosis in Hamadan Province, west of Iran: A Population Based Cross Sectional Study (2005-2013) 

**Published:** 2016-09-20

**Authors:** Meysam Olfatifar, Manoochehr Karami, Seyed Mehdi Hosseini, Masoud Parvin

**Affiliations:** ^a^ Student Research Committee, Hamadan University of Medical Sciences, Hamadan, Iran; ^b^ Research Center for Health Sciences , Hamadan University of Medical Sciences, Hamadan, Iran; ^c^ Department of Epidemiology, School of Public Health, Hamadan University of Medical Sciences, Hamadan, Iran

**Keywords:** Epidemiology, Spatial Analysis, Pulmonary Tuberculosis

## Abstract

**Background:** This study was conducted to detect clusters of pulmonary TB cases in Hamadan Province, west of Iran.

**Methods:** All patients with pulmonary tuberculosis recorded in the surveillance system from 2005 to 2013 were studied. The spatial scan statistic was used to detect significant clusters in status of unadjusted and adjusted for age, sex and location residence variables.

**Results:** Clusters with high rate for both purely spatial and space-time analyses were seen in the same geographical areas composed of four city of Asadabad, Bahar, Toyserkan and Nahavand. Adjustment for mentioned variables did not change location of detected clusters with high rates.

**Conclusions:** Findings revealed evidence of significant clusters in Hamadan Province. Study results may help the health system to develop effective public health interventions and extend preventive interventions. However more study are needed to better explain of detected clusters due to limited access to effecting factors.

## Introduction

Tuberculosis is a top infectious disease killer worldwide and nearly one-third of the world's people are infected with latent tuberculosis^1^. Each year, about nine million of new cases of TB occurred in the world. Moreover, TB is responsible for nearly 1.7 million deaths annually^2^.

In 1993, WHO declared TB as a global emergency and called all countries to control TB^3^. Despite of disease agent‏ identification, vaccine and highly effective drugs, tuberculosis is still a major health challenges globally^4^, because multidrug resistance tripled from 2009 to 2013 and in 2013,‏ 48% of TB patients worldwide had a documented HIV test result^5^. According to an estimate in 2014, 480000 people have been infected with multidrug-resistant tuberculosis^1^. However, one way to reduce drug resistance in Iran with respect to its neighbors is establishment of medical campus in boundary areas^6^.

Although the distribution of TB can be described by some known factors at the individual level^7^, growing evidence underlying factors (Physical environment, contact patterns and population density) are good predictor of existing models^8,9^. TB control activities should focus on facilitating strategies to access vulnerable populations with low access to health services where transmission of tuberculosis remained as a public health problem^10^. The first step for such studies is to explain the spatial pattern of TB using geographic information system (GIS) and spatial analysis used for census tracts, postal areas and urban blocks^11^.

Although there are some studies on the epidemiology of TB in the Hamadan Province^12^, but this study is the first that investigated spatial distribution and clusters of the disease.

## Methods

In this cross-sectional study, data of patients with TB including age, gender, location, type of TB and diagnosis date from January 2005 to December 2013 were taken from TB surveillance systems at provincial level. Population data for 2006 and 2011 were obtained from census which officially published by Statistical Center of Iran. Population and participants' data were used to build required file to run SaTScan software. We used aggregate data instead of individual data. Accordingly, there was no need to ask participants to complete the informed consent.

Distribution of age and sex and incidence rate of pulmonary TB cases for each city was calculated using Stata software Ver.11. Pulmonary TB cases for each city as the numerator and the average population of two censuses for each city were used as denominator. Then SaTScan software ver. 9.3 was run to analytical analysis. Spatial scan statistic was used to detect purely spatial and space-time cluster with discrete Poisson distribution. This method of analysis is used in situation that number of cases in area according to a known underlying population at risk have Poisson distributed. Spatial scan statistic imposed cylindrical into the map and stand in turn on the coordinate center of each area that will be allocated using GIS and its radius and height changed anywhere from zero to the upper limit determined by the investigator. Moreover, generated numerous cylinders were tested using Monte Carlo simulations based on the null hypothesis. The maximum spatial cluster size once was considered 50% ≤ population at risk and once again, to detect smaller clusters was considered 25% ≤ population at risk. Purely spatial and space-time cluster analysis were done to detect clusters with high rates and low rates. All analyzes were done twice, once unadjusted for age, sex and residence location of cases and once again adjusted for those. Number of the simulation was 999 times. *P* values less than 0.05 were considered statistically significant.

## Results

During nine year of the study period, 1057 human TB cases were reported to provincial surveillance system. Of them, 67.3% (712 cases) were pulmonary TB. Among them, 364 cases (51.1%) were male and 48.9% were female. Mean age of cases totally was 57.41 ± 21.47 yr and 59% and 41% of cases residence in urban and rural areas, respectively. Average annual incidence of TB in Hamadan Province was 2.08 cases per 10,000 in the study period. Kabudrahang and Razan cities with 3 and 1.56 case per 10,000 had the highest and lowest incidence rates, respectively (Table 1).

**Table 1:** Cumulative incidence of pulmonary TB cases for each county between 2005 and 2013 on average

**City**	**Number of cases**	**Population** **at risk**	**Incidence** **per 10,000/yr**
Asadabad	54	213,034	2.53
Bahar	55	246,123	2.23
Hamadan	270	1,289,125	2.09
Kabudrahang	86	285,816	3.00
Malayer	114	578,179	1.97
Nahavand	60	362,760	1.65
Razan	36	229,380	1.56
Toyserkan	37	214,613	1.72
Total	712	3,419,030	2.08

One most likely cluster was detected with regard to the maximum length of ≤50% at-risk populations and high rates of TB. This cluster had composed of four city of Asadabad, Bahar, Toyserkan and Nahavand (Table 2). In order to detect smaller clusters in study area similar analysis with a maximum length of ≤25% population at-risk with high rates of tuberculosis was performed for search window that detected cluster in same location. After adjustment for age, sex and residence location variables location of detected cluster did not change but there was difference in characteristics related to each cluster (Table 2). Analysis with low rate did not detect a cluster.

**Table 2:** Primary purely spatial clusters with higher and lower rates of TB in Hamadan Province using search window maximum length of≤50% and≤25% risk population, before and after adjustment for age-gender and residence location

**Adjustment**	**Center coordinates**	**Radius (km)**	**No. of cases**	**Expected number** **of cases in clusters**	**Relative risk** **of clusters**	**Log Likelihood** **Ratio**	***P*** **value**
Unadjusted	34.778N-48.028E	64.99	205	70.60	3.67	99.301	0.001
Adjusted	34.778N-48.028E	64.99	205	103.98	2.36	47.039	0.001

Using the search window with a maximum length of ≤50% at-risk population with high rates of TB The most likely cluster or cluster with high probability again had composed of four city Asadabad, Bahar, Toyserkan and Nahavand during the years 2008 to 2012 (Table 3). Search window with a maximum length of ≤25% was used to detect significant clusters that detected cluster with same geographic of the first location. In this case, location of detected cluster was the same for both unadjusted and adjusted status but there was difference in characteristics related to each cluster (Table3) and time of cluster after adjustment was from 2008-2011.

**Table 3:** Primary Space-time clusters with higher and lower rates of TB in Hamadan Province using search window maximum length of≤50% and≤25% risk population, before and after adjustment for age-gender and residence location

**Adjustment**	**Center coordinates**	**Radius (km)**	**No. of cases**	**Expected number** **of cases in clusters**	**Relative risk** **of clusters**	**Log Likelihood** **Ratio**	***P*** **-value**
**High rates**							
Unadjusted	34.778N-48.028E	64.99	130	41.57	3.60	65.898	0.001
Adjusted	34.778N-48.028E	64.99	109	51.68	2.31	26.585	0.007
**Low rates**							
Unadjusted	34.542N-48.332E	49.05	0	16.81	0.00	17.000	0.033
Adjusted	34.322N-48.793E	0.00	9	10.51	0.85	0.1153	1.000

In addition, the cluster size of ≤50% was used to scan areas with low rate of TB, that a cluster was discovered and had composed of Toyserkan, Nahavand, Asadabad, Malayer and Bahar cites in 2014 and was not significant but in adjusted status detected cluster had formed of Malayer City in 2006 (Table 3).

## Discussion

The spatial and temporal distributions of pulmonary tuberculosis cases were studied. One significant spatial cluster and one significant space-time cluster was detected. Location of both purely spatial and space-time clusters with high rates was in the same geographical areas and had composed of four city Asadabad, Bahar, Toyserkan and Nahavand, the time period of space-time clusters with high rates before and after adjustment for age, sex and residence location of cases were from 2008-2012 and 2008-2011. Covariates adjustment for both purely spatial and space-time analysis did not change location of high rates detected clusters but changed the characteristic of cluster. Perhaps we can infer that the adjusted variables somewhat explain the existence of significant clusters, because the likelihood ratio and RR of clusters declined after adjustment.

Studies to cluster TB in Africa and to cluster Smear-Positive TB in Ethiopia location of both purely spatial and space-time clusters were in the same geographical areas consistent with other studies ^13,14^. The distribution of TB in areas was not randomized and with a special pattern have been forming cluster.

The limitations of this study were inadequate access to factors related‏ to clusters distribution due to inefficiency surveillance system in the whole country and one was, excluding twelve TB cases of the Famenain City from study due to the recent separation of the city from Hamadan Province and the lack access to appropriate map. The time and space clusters investigation may have an important role in public health policy ^15^ and the systematic use of this method in TB care system can lead to better management of the financial and human resources, and ultimately better control of TB. Furthermore our results may be a framework for other studies of mostly etiology aspect, until investigate in field of environmental factors, socioeconomic factors, host factors and specific microorganisms responsible for clustering in areas with high rates of TB through active TB case finding. For example, the impact of environmental factors on the geographical distribution of TB was studied in Khuzestan Province and showed that the distribution of TB was influenced by environmental factors^16^. The impact of other affecting factors on incidence of the TB, such as low educational level^17,18^age, poverty^18^, individual factors^19^ immigration ^20^ and crowded households^21^ have been studied in other areas of the world.‏

## Conclusions

Findings revealed evidence of significant clusters in Hamadan Province. Study results might be useful to develop effective public health interventions and extend prevention interventions. However, further studies are needed to explain better the clusters regarding to limit access to effecting factors, because location of detected clusters may change after adjustment for associated factors.

## Acknowledgments

The study was funded by Student Research Committee affiliated to Vice-chancellor for Research and Technology, Hamadan University of Medical Sciences (No. 931212).

## Conflict of interest statement

Authors have no conflict of interests.

## Highlights

- Evidence indicates existence of significant cluster of pulmonary TB in Hamadan Province, west of Iran.
- Clusters with high rate for both purely spatial and space-time cluster analysis were seen in the same geographical areas.
- Significant clusters were detected in the cities of Asadabad, Bahar, Toyserkan and Nahavand
